# Phospholipase D, a Novel Therapeutic Target Contributes to the Pathogenesis of Neurodegenerative and Neuroimmune Diseases

**DOI:** 10.1155/2024/6681911

**Published:** 2024-03-07

**Authors:** Weiwei Zhang, Feiqi Zhu, Jie Zhu, Kangding Liu

**Affiliations:** ^1^Neuroscience Center, Department of Neurology, The First Hospital of Jilin University, Changchun, China; ^2^Cognitive Impairment Ward of Neurology Department, The Third Affiliated Hospital of Shenzhen University Medical College, Shenzhen, China; ^3^Department of Neurobiology, Care Sciences and Society, Karolinska Institute, Karolinska University Hospital Solna, Stockholm, Sweden

## Abstract

Phospholipase D (PLD) is an enzyme that consists of six isoforms (PLD1–PLD6) and has been discovered in different organisms including bacteria, viruses, plants, and mammals. PLD is involved in regulating a wide range of nerve cells' physiological processes, such as cytoskeleton modulation, proliferation/growth, vesicle trafficking, morphogenesis, and development. Simultaneously, PLD, which also plays an essential role in the pathogenesis of neurodegenerative and neuroimmune diseases. In this review, family members, characterizations, structure, functions and related signaling pathways, and therapeutic values of PLD was summarized, then five representative diseases including Alzheimer disease (AD), Parkinson's disease (PD), etc. were selected as examples to tell the involvement of PLD in these neurological diseases. Notably, recent advances in the development of tools for studying PLD therapy envisaged novel therapeutic interventions. Furthermore, the limitations of PLD based therapy were also analyzed and discussed. The content of this review provided a thorough and reasonable basis for further studies to exploit the potential of PLD in the treatment of neurodegenerative and neuroimmune diseases.

## 1. Introduction

Phospholipases constitute a class of enzymes that catalyze glycerophospholipids in organisms, and widely present in animals and plants [1]. Phospholipase D (PLD) was first discovered in plants by Hanahan and Chaikoff in 1947 [2], and in 1973, Saito and Kanfer [3] first reported its role in mammals. Subsequently, advancements in biochemistry and molecular biology have widely cloned various PLD subtypes (PLD1, PLD2, and PLD3). The mammalian PLD superfamily has gained prominence for its catalytic role in the hydrolysis of phosphatidylcholine (PC), the most abundant phospholipid in membrane phospholipids, into choline and phosphatidic acids (PAs), which serves as a second messenger signal [4]. The signal-dependent activation of PLD has been observed in various neuronal cell types, including neurons, glial cells, and glioma cells, and various neurotransmitters and neuromodulators have been shown to activate PLD [5]. The omnidirectional interaction between different types of central nervous system (CNS) cells is crucial for the dynamics of CNS function. PLD has emerged as one of the most significant communication mediators. PLD may influence essential cellular processes, such as actin cytoskeletal reorganization, vesicular trafficking, and signal transduction, which can potentially contribute to pathological roles in the brain [6, 7]. Considering its various characteristics and effects on brain function, PLD has become a target for CNS therapeutic intervention. In this review, a literature search was conducted from 2010 to 2023 using the following keywords: PLD, PLD inhibitor, Alzheimer disease (AD), Parkinson's disease (PD), amyotrophic lateral sclerosis (ALS), spinocerebellar ataxia (SCA), multiple sclerosis (MS), and therapeutic target. This review presents an overview of the current understanding of the six human PLD isoenzymes, their structures, their roles in both physiological and pathological processes of brain development, the relationship between PLD and neurodegenerative and neuroimmune diseases, as well as tools for studying PLD therapy and PLD inhibitors. The review provides a possibility for further understanding the therapeutic potential of PLD in neurodegenerative and neuroimmune diseases.

## 2. Features of PLD

**Figure 1:**
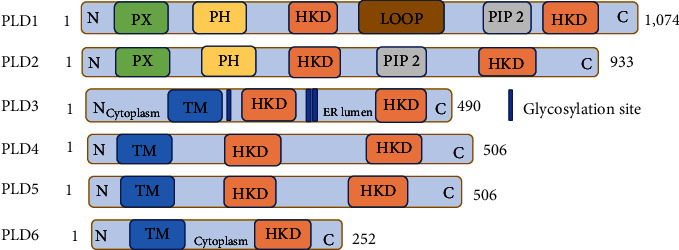
Cartoon schematic of mammalian PLDs (PLD1–PLD6).

PLD enzyme activity has been universally described in almost all organisms, and has formed a superfamily that exists in bacterial, viral, mammalian, and plant cells. PLD was initially cloned from castor oil, and sequence information has enabled other organizations to clone PLD enzymes from various organisms [8]. Almost 3 decades after the first description of PLD in plants, Saito and Kanfer [3] were the first to demonstrate PLD activity in mammalian tissues by partially purifying PLD from rat brain. More than 4,000 PLD sequences are archived in the GenBank of the US Biotechnology Information Center [9]. An important feature of the PLD protein family members is that they have two subdomains composed of the same amino acid sequence (HXKX4DX6GSXN), called the HKD motif [10]. However, there are exceptions, some PLDs do not have this subunit and some have only one HKD subdomain, thus, the PLD family can be classified as follows: (1) active phospholipase with HKD subunit; (2) phospholipase with HKD subunit but lacking lipase activity; and (3) phospholipase without HKD subunit (Figure 1). In mammals, there are two canonical PLD isoforms, PLD1 and PLD2, which share 57% amino acid conservation. Both isoforms have a conserved C-terminus, two HKD catalytic domains that combine to form a single active site, and conserved tandem PX- and PH-domains [11, 12]. Although PLD3 (also known as Sam-9 or HUK4), PLD4, PLD5, and PLD6 (also known as mitochondrial PLD, MitoPLD, Zucchini, or Zuc) possess HKD domains or variations, they are considered nonclassical PLDs because they lack the PX- and PH-domains as well as the traditional PLD activity required to convert PC to PA [7]. Single-stranded nucleic acid exonucleases PLD3 and PLD4 control endosomal nucleic acid sensing [13, 14]. Due to the lack of histidine, lysine, and the first catalytic motif's histidine as well as the second's histidine, PLD5 is most likely catalytically inactive, resulting from insufficient conservation of the catalytic domains [15]. PLD6, which encodes a single protein with the HKD motif, hydrolyzes cardiolipin to PA and does not demonstrate any ribonucleic acid or deoxyribonucleic acid nuclease activity in vitro (Table 1) [20].

**Figure 2:**
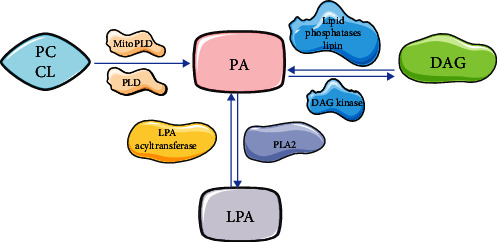
Signaling lipid pathway.

**Figure 3:**
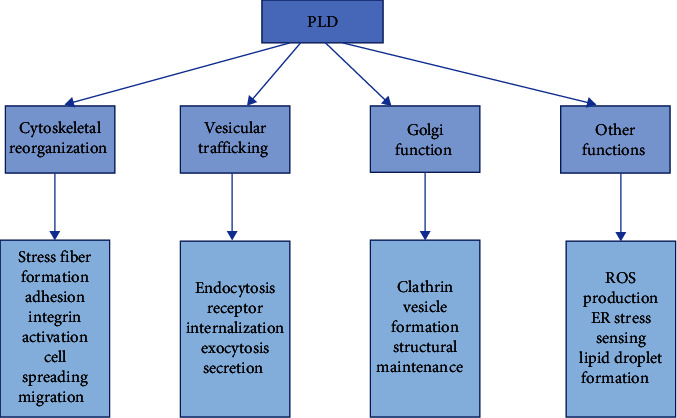
Illustrations of PLD function in mammals.

PLD family is a transphosphatidylase that catalyzes the exchange of head groups on phosphodiester bonds linking various substrates (Figure 2). First, PLD uses water as a nucleophile to hydrolyze phospholipid substrates, such as PC, to produce the membrane lipid PA, generate soluble choline in the cytosol [21]. Second, peripheral members of the superfamily can apply other phospholipid substrates, such as cardiolipin, to release PA or protein deoxyribonucleic acid bonds caused by stalled topoisomerase 1 or hydrolyze the phosphodiester bonds found in the deoxyribonucleic acid backbone [22]. Third, PLD1/2 can use nucleophiles other than water to generate new lipids by exchanging complex heads for simple head groups on phospholipid substrates. In the presence of ethanol, their enzymes mediate the phosphatidylation process, where the phosphoinositide group in PC is transferred to ethanol instead of water, resulting in the formation of phosphatidylethanol with consumed PA [23]. PLD and PA regulate a wide range of cellular processes, including vesicle trafficking, endocytosis, phagocytosis, stress responses, pathogen resistance, and apoptosis, etc., which are all closely related to the normal function of brain cells [24, 25] (Figure 3).

## 3. Roles of the PLD Pathway in Regulating Brain Function

### 3.1. PLD in Mammalian Target of Rapamycin Signaling

The serine/threonine kinase known as mammalian target of rapamycin (mTOR), regulates cell growth and metabolism in response to stress, growth stimuli, and nutrition [26]. mTOR activity has been associated with PLD and PA [27]. PA containing two saturated fatty acids, such as dipalmitoyl-PA, causes the mTORC2 complex to disintegrate, while PA containing palmitate (saturated) and oleate (monounsaturated) stabilizes both mTORC1 and mTORC2 in the presence of PLD suppression [28]. This suggests that PLD provides PA in the appropriate format for mTOR stabilization. It has been proposed that PLD, particularly PLD1, may contribute to the activation of mTOR due to PLD is located in the lysosomes [29]. This is due to the lysophosphatidic acid acyltransferase pathway generates PA in the endoplasmic reticulum, which is then transported to other cellular locations through vesicular trafficking [30]. PLD has been suggested to play a role in mTOR signaling in relation to CNS disorders.

### 3.2. PLD and Ras Signaling Pathway

The Ras superfamily is a group of proteins known to directly activate PLD [31]. The first guanosine triphosphatases (GTPases) to be reported as PLD activators are ADP-ribosylation factor (Arf) and adenosine diphosphate-ribosylation factor-like protein 2 (Arl2) [32]. Arf is found to be a cytosolic factor necessary for GTP*γ*S-dependent stimulation and capable of activating PLD in HL60 cell membranes [33]. Inhibition of Arf with the drug brefeldin A or overexpression of dominant negative Arf1 or Arf6 blocks PLD activation [34]. Recombinant Rhodopsin (Rho), recombinant cell division cycle protein 42, and Ras-related C3 (Rac) botulinum toxin substrate 1 are proposed to be selective binding activators for PLD1, enhancing substrate binding affinity [31]. Pretreatment with Rho GTPase inhibitors such as *Clostridium difficile* or *C. botulinum* C3 toxin prevents PLD activation [35]. PLD affects Rac activity by producing PA, which facilitates the dissociation of the Rho-specific guanine nucleotide dissociation inhibitor (Rho GDI) and promotes Rac1/2 plasma membrane interaction [36]. Through their C-terminal PA-binding polybasic motifs, PA further attracts and stabilizes the engagement of Rho family guanine nucleotide exchange factors dedicator of cytokinesis 1/2 and T-lymphoma invasion and metastasis-inducing protein 1 with the plasma membrane [37].

### 3.3. PLD and Mitogen-Activated Protein Kinase Signaling Pathway

By signaling through their specific receptors, growth factors, hormones, and chemokines activate the mitogen-activated protein kinase (MAPK) pathway, which in turn activates various protein kinases [38]. PLD and PA are closely associated with several steps in the MAPK pathway, and PLD overexpression has been linked to increased extraneous signal-regulated kinase (ERK) activity, as evidenced by higher transcription of genes downstream of ERK-activated transcription factors, including signal transducer and activator of transcription 3 [39]. By regulating receptor endocytosis, PLD may have an indirect effect on ERK activation [40]. It has been demonstrated that PLD activity controls the process of receptor endocytosis for a range of cell surface receptors, including G protein-coupled receptors (GPCRs) such as the angiotensin II receptor and the *μ*-opioid receptor, as well as receptor tyrosine kinases like epidermal growth factor receptor [41].

### 3.4. PLD and Sphingolipid Signaling Pathway

**Figure 4:**
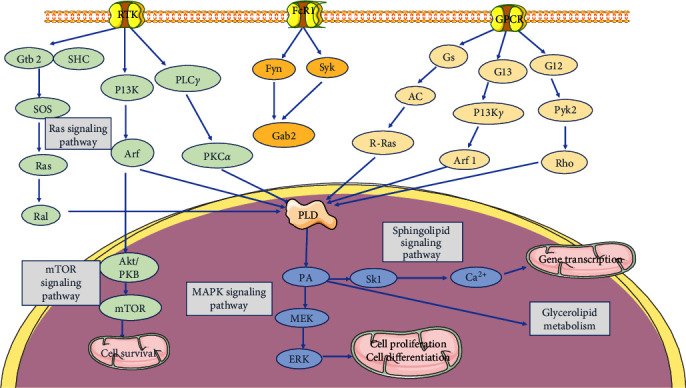
Phospholipase D signaling pathway.

Sphingosine kinase is the enzyme responsible for producing sphingosine-1-phosphate (sph-1-P) in response to various stimuli, such as growth factors, cytokines, and agonists of GPCRs [42]. A number of studies suggest that sphingolipid signaling can be a promising novel target for neuroprotection, aiming to counteract the pathophysiology of CNS disorders related to oxidative stress, mitochondrial dysfunction, cell apoptosis, and lipid hydrolysis [43, 44]. Sph-1-P is the primary regulatory lipid involved in PLD metabolism and has been implicated in the regulation of various aspects of cell physiology, such as mitogenesis, differentiation, migration, and apoptosis [45, 46]. We summarize the schematic diagram of the PLD signal pathway in Figure 4.

## 4. Roles of PLD in Neurodegenerative and Neuroimmune Diseases

### 4.1. PLD and AD

AD is a neurodegenerative condition clinically characterized by progressive memory impairment, compromised cognitive abilities, altered and inappropriate behaviors, as well as diminished language skills [47]. PLD1 expression and activity are elevated in brain tissue of AD patients compared to healthy controls, particularly within the caveolar membrane fraction [48]. Interestingly, PLD1 physically interacts and colocalizes with amyloid precursor protein (APP) and caveolin-3 [49]. One study discovered that APP correlates with the structural pleckstrin domain of PLD1 homolog, and that the amyloid region of APP interacts with PLD, suggesting that the upregulation of PLD1 may play a role in AD associated neuronal pathology [50]. Bourne et al. [51] reported that an abnormal increase in neuronal PLD1 is crucial for oligomeric amyloid formation, which can lead to synaptic dysfunction and potential memory deficits. The most intriguing study has centered on PLD2. PLD2 is activated by amyloid *β* (A*β*)—peptide in neurons, PLD2 ablation rescues memory deficits and confers synaptic protection in AD mouse models [52]. Recently, whole exome sequencing of 14 large families of late-onset AD revealed that the rare coding variant in PLD3 (*PLD3 p.V232M*) increased the risk of AD [53]. In a subsequent meta-analysis, *PLD3 p.V232M* variant was found to contribute to the risk of attention deficit disorder, but its effect was smaller than initially reported and comparable in magnitude to that of the apolipoprotein E-*ε*4 allele [54]. The PLD3 level in human prefrontal cortex was found to be inversely associated with the severity of A*β* pathology as well as the rate of cognitive decline in 531 participants enrolled in the aging project [55]. Accumulation of A*β* in the brain mediates various aspects of the pathogenesis of attention deficit disorder, and A*β*-stimulated PLD activity correlates with lactate dehydrogenase release, an indicator of cell death, suggesting that the neurotoxic effects of amyloid are mediated by PLD [50]. PLD1/2 is also involved in APP and presenilin trafficking and it is crucial for APP metabolism and secretion [56]. In cultured neurons, PLD2 is activated by A*β*, and reduced PLD2 levels prevent A*β*-induced PLD activation [57]. PLD activity was elevated in an AD transgenic mouse model, and PLD2 deletion hindered the synaptotoxicity of A*β*42 oligomers [52]. Consequently, PLD1/2 not only mediates downstream A*β* signaling and modulates A*β* receptor availability at the synapse but also impacts A*β* binding to related receptors [58]. As a novel target, PLD may represent a promising therapeutic option for the management of AD.

### 4.2. PLD and PD

Dopamine levels in the striatum and substantia pars compacta are decreased in PD [59]. Synuclein, a protein known to play a pathogenic role in PD, interacts with PLD2 [60]. Both PLD1 and PLD2 immuno-precipitate with the neurotoxic synuclein peptide, and PLD2 is inhibited in vitro by synuclein [61]. In human dopaminergic cells, activation of PLD2 by the muscarinic receptor is thought to be associated with a loss of synuclein's PLD2 inhibitory effects [62]. Dopamine neurons in the rat substantia nigra pars compacta will rapidly degenerate when PLD2 is overexpressed, and synuclein can inhibit this process [63]. Further evidence of compromised autophagic mechanisms comes from the accumulation of autophagic vesicles in the cytoplasm of neurons in PD brains [64]. More recent study has shown that pharmacological reduction of PLD1 disrupts autophagic flux and induces synuclein aggregation [65]. When PLD1 activity was decreased, accumulation of the microtubule-associated protein light chain 3, prostacyclin, and polyubiquitinated proteins suggested a problem with autophagic flux [65, 66]. Decreased autophagy flux and accumulation of synuclein clumps in autophagosomes were seen in PD-associated cells [67]. To cure neurotoxicity caused by synuclein accumulation, PLD1 was overexpressed. PLD1 plays a key function in modulating the generation of autolysosomes, which supports the maintenance of autophagic flux [68]. A decline in PLD1 lead to impaired clearance of synuclein aggregation [69]. Further studies will deepen our understanding of how increased PLD expression leads to the progression of PD.

### 4.3. PLD and Amyotrophic Lateral Sclerosis

Amyotrophic lateral sclerosis (ALS) is a progressive neuromuscular disease characterized by the failure of both lower and upper motor neurons [70]. According to Kankel et al.'s [71] study, the pathway controlling PLD activity is a crucial regulator, as supported by data from mouse and human studies. In the mouse model of ALS, there is a corresponding increase in PLD1 levels, which correlates with early-onset ALS in postmortem human tissues [72]. In the drosophila model of ALS, the downregulation of PLD improves degenerative characteristics [73]. Lacoangeli et al. [74] conducted a meta-analysis using a published study of postmortem gene expression in motor neurons from ALS patients, suggesting that the PLD1 pathway may play a role in regulating the ALS phenotype. This study identified 41 genes with high levels of ribonucleic acid expression associated with early disease onset, including PLD1. Additionally, v-ral simian leukemia viral oncogene homolog B and adenosine diphosphate-ribosylation factor GTPase activating protein 3 are components of the PLD1 signaling network [74]. PLD inhibitor may be useful for ALS therapy by inhibiting both PLD1 expression and activity.

### 4.4. PLD and MS

MS is a neurological autoimmune disease that causes permanent disability, where the myelin sheath is attacked by the autoimmune response [75]. PLDs are well-established as they are crucial for neuronal cell neurite outgrowth, particularly axon outgrowth [76]. Currently, one of the main areas of interest for MS treatment is the inhibition of lymphocyte trafficking [77]. PLD1 has been identified as a cell mobility regulator. The absence of PLD1 in vitro models resulted in decreased blood–brain barrier and chemokine-induced lymphocyte static adherence to intercellular adhesion molecule 1 and vascular cell adhesion molecule 1, as well as reduced cell migration and motility [78]. The disease severity was reduced in experimental allergic encephalomyelitis (EAE) mice lacking PLD1 [79]. Ahn et al.'s [80] study also showed that PLD1, primarily composed of ED1-positive macrophages and glial fibrillary acidic protein-positive astrocytes, significantly increased in the spinal cord during the peak of EAE. These findings show that PLD1 is elevated during CNS autoimmune inflammation and may play a role in macrophage and astrocyte activation in EAE related lesions.

### 4.5. PLD and Spinocerebellar Ataxia

A rare progressive neurodegenerative disease known as spinocerebellar ataxia (SCA) is characterized by a loss of coordination and balance [81]. Through whole exome sequencing, Nibbeling et al. [53] discovered new genes, including PLD3, in patients with autosomal dominant SCA. PLD3 was found in the endoplasmic reticulum, as demonstrated by functional studies, the *p. L308P* missense mutation in PLD3 might lead to a loss of function that reduced phospholipase activity in COS-7 cells and cause endoplasmic reticulum stress [53]. They speculated *PLD3 p. L308P* might be a new pathogenic site for SCA [53]. We summarize the studies on the relationship between PLD and neurodegenerative and neuroimmune diseases in Table 2.

## 5. Tools for Targeting PLD Therapy and PLD Inhibitors

### 5.1. PLD Knockout Mice Model

Surprisingly, it has been observed that mice lacking one or both isoforms are viable and exhibit normal phenotype [87]. PLD inhibitors can be useful therapeutic agents without posing a significant risk to health. In the event that one isoform is absent, the other's expression does not increase, and mice without PLD1 or any other PLD subtype show normal development, good health, fertility, and overtly normal behavior [88]. Nonetheless, there are phenotypes associated with the absence of PLD. Mice with a single or double PLD1/PLD2 deletion exhibit signs of poor brain development, with smaller brains at 14–27 days after birth [89]. PLD1/PLD2 knockout mice also showed impaired cognitive function in terms of social cognition and object recognition [90]. Brain microdialysis of PLD1/PLD2 single knockout mice showed significantly reduced hippocampal acetylcholine (Ach) release after behavioral stimulation. This could be due to decreased choline production resulting from reduced PLD activity, as choline is a precursor for Ach formation [91]. These findings may be relevant to the cognitive impairment observed in AD. In a transgenic AD model, PLD2 knockout mice demonstrated protection against the synaptotoxic and memory-impairing effects of *β*-amyloid [52]. PLDs may possess nonphospholipase functions, such as scaffolding, which complicates the interpretation of the knockout animal abnormalities [92]. As a result, their absence could possibly disrupt multiprotein complexes.

### 5.2. Typical Tools Used to Target PLD Therapy

The most valuable resources for investigating PLD function are the molecular inhibitors that have been identified. Considering the potential for any medication to have unforeseen off-target effects, these approaches complement each other. Primary alcohols remain the most frequently employed PLD inhibitor [93]. The majors decision was made due to the finding that primary alcohols exhibit significantly higher nucleophilicity compared to water [94]. However, the observation that the concentration of alcohol required to substantially inhibit PA production by PLD results in severe cytotoxicity compared to the lower concentrations commonly used in the previous study [7]. Target validation with selective small compounds is essential for PLD inhibition to become a viable treatment method [88]. The discovery that the neuropsychiatric medication halopemide is a potent PLD inhibitor lead to the development of a series of pharmaceutic papers published between 1980 and the mid-2000s [95]. An analog named 5-fluoro-2-indolyl des-chlorohalopemide is a powerful inhibitor of PLD1/2, and its half-life and bioavailability have facilitated its extensive use in cell culture and animal studies [96]. 5-Fluoro-2-indolyl des-chlorohalopemide has so far mimicked the effects seen in PLD1/2 knockout animals [96]. It is interesting to note that halopemide, which was developed to treat psychosis due to its ability to block dopamine receptors, was clinically used at a high enough dose to completely inhibit PLD activity [97]. This suggests that even long-term use of PLD inhibitors does not lead to unacceptably severe toxicity.

### 5.3. PLD Inhibitory Proteins

Munc-18-1, a syntaxin-binding protein more abundant in neurons, is crucial for the exocytosis of synaptic vesicles. Munc-18-1 directly interacts with the phox homology structural domains of PLD1 and PLD2 to inhibit PLD activity in vitro [98]. In addition to Munc-18-1, it has been demonstrated that clathrin assembly protein 3, extracted from the cytoplasm of rat brain and abundant in neural tissues, also reduced PLD1 activity in vitro [99]. Similar to assembly protein 3, amphiphysin I and II have been identified in rat brain cells. They inhibit phorbol-12-myristate-13-acetate-induced PLD activity and block PLD1/2 activity when they are overexpressed in cells [100]. The precise physiological significance of PLD suppression by these synaptic vesicle proteins remains unknown, however, it is likely that they can hinder PA synthesis during the early stages of vesicle formation. In addition to the PLD inhibitors associated with vesicles and actin, several other proteins that do not fit into any specific category have also been identified as PLD inhibitors. It was discovered that a cytosolic component called aldolase directly inhibits PLD2 [101]. Although the physiological significance of the aldolase–PLD2 interaction is unknown, it may assist PLD in carrying out its role in regulating cellular bioenergetics. The G subunit from heterotrimeric G proteins is another protein that has been shown to inhibit PLD. Since N-terminal PLD truncation mutants are found to be resistant to G inhibition, it is seemingly that recombinant G protein inhibits PLD1/2 activity in vitro by interacting with the PH structural domain [102].

### 5.4. PLD Isoenzyme Inhibitors

**Figure 5:**
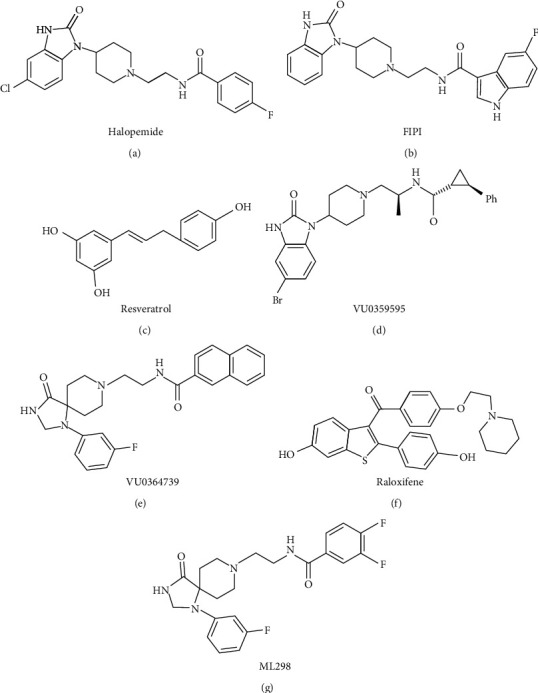
Chemistry formulas of tools for studying PLD function and emerging small PLD inhibitors.

The field has not progressed significantly despite efforts dating back to the 2000s to identify small molecules capable of modulating the function of specific PLD enzymes either directly or indirectly (e.g., steroid products such as pancreatic lactones or polyphenolic natural products like resveratrol, or direct mimetic phosphate compounds like tungstate). This is partially due to the fact that these ligands appear to lack specificity [103]. The discovery of PLD inhibitors with isozyme selectivity improves drug metabolism and adjuvant pharmacology, making them suitable for in vivo proof-of-concept investigations. Brown's group initiated an optimization strategy using halopemide as the foundation. One example of a diversity-oriented synthesis strategy that yielded the first highly PLD1 selective inhibitors is VU035959592. Notably, compared to 5-fluoro-2-indolyl des-chlorohalopemide and halopemide, VU0359595 demonstrated a favorable pharmacokinetics profile and significantly improved ancillary pharmacology, enabling in vivo proof-of-concept studies. Remarkably, VU0364739, the first highly selective PLD2 inhibitor, has the ability to penetrate the brain, while VU0359595, which prefers PLD1, can only enter peripheral tissues. Brown's group assessed numerous non-N-aromatic compounds unconnected to nitrogen in an attempt to create a PLD2 inhibitor comparable to ML298 but with improved CNS penetration. This strategy was selected due to the triazaspirone core's ability to provide a variety of PLD pharmacological properties. As a result of this process, they discovered ML395, a pyridylmethyl congener with potent inhibition of PLD292. Prior to this, raloxifene was the only selective estrogen receptor regulator chemotype that could potentially inhibit structurally and phylogenetically distinct PLD enzymes. This marked the first step towards the potential universal inhibition of PLD enzymes with different structures and phylogenies [104]. The creation of these substances has made it possible to develop a novel set of tools for examining the general activity of PLD enzymes and the function of specific PLD isozymes in a range of neurodegenerative and neuroimmune diseases (Figure 5).

## 6. Conclusion

Activities have been identified in all genera since the discovery of PLD activity in plant tissue. Since then, advancements in scientific methods have led to the cloning of PLD genes, the identification of signature motifs, and a deeper understanding of the structure and catalytic mechanism of PLD. PLD knockout mouse models have enabled us for the first time to examine the functions of PLD in the context of the entire organism. Here, we review numerous studies demonstrating PLD's role in the pathogenesis of neurodegenerative and neuroimmune disorders. PLD may be related to CNS immunological and degenerative processes in at least four different ways. The first one concerns PLD and its possible association with *α*-synuclein; the second identifies PLD isozymes as caspases' substrates; the third discusses how changes in PA metabolism can affect membrane dynamics and affect cell viability; and the fourth one deals with PLD's role in survival pathways in relation to mTOR. The prospect that PLD proteins can serve as drug targets is getting closer to reality as our knowledge of PLD's involvement in the pathophysiology of these CNS illnesses and the potential of PLD for medication becomes more refined.

## Figures and Tables

**Table 1:** Characteristics of different PLD subtypes.

	PX/PH domains	Base numbers	Intracellular localization	Isoform-specific tissue expression	References
PLD1	Exist	1,074	Perinuclear vesicular localization, plasma membrane	Heart, brain, pancreas, uterus, and intestine	[16]
PLD2	Exist	933	Plasma membrane	Brain, placenta, lung, thymus, prostate, and uterine tissue	[17]
PLD3	Null	490	The luminal side of the endoplasmic reticulum	Brain, smooth muscle, skeletal muscle, heart muscle, lung tissue, and epididymis	[18]
PLD4	Null	506	Endoplasmic reticulum and golgi apparatus	Liver, spleen, brain, and lymph nodes	[19]
PLD5	Null	506	Endoplasmic reticulum and golgi apparatus	Liver, spleen, brain, and lymph nodes	[15]
PLD6	Null	252	The outer surface of the mitochondria	Adrenal glands and gonads	[20]

PLD, phospholipase D.

**Table 2:** Studies on the relationships between PLD and neurodegenerative and neuroimmune diseases.

Study subjects	PLD subtypes	Main findings	Countries/years of publications	References
AD mouse model	PLD3	PLD3 affected axonal spheroids and network defects in AD	The USA/2022	[82]
AD mouse model	PLD3	PLD3 was associated with *β*-amyloid plaques and cognitive function in AD	The USA/2021	[83]
AD patients	PLD1	Elevated PLD1 in AD patients' hippocampus was relevant with synaptic dysfunction and memory deficits	The USA/2018	[58]
AD *C. elegans* model	PLD1	PLD functional ablation had a protective effect in an AD *C. elegans* model	Portugal/2018	[84]
PD cell model	PLD1	PLD1 downregulation might constitute an early mechanism in the initial stages of neurodegeneration	Spain/2018	[85]
PD patients	PLD1	PLD1 modulated *α*-synuclein toxicity	China/2022	[69]
PD mouse model	PLD2	The lipase activity of PLD2 was responsible for nigral neurodegeneration in a rat model of PD	Spain/2018	[85]
MS patients	PLD1	PLD1 could be used as putative biomarkers for evaluation of therapeutic responses to IFN-*β* in MS patients.	Iran/2017	[86]
ALS mouse model	PLD1/2	PLD1/2 inhibitor could improve ALS phenotype	The USA/2022	[73]
SCA patients	PLD3	PLD3 might be a novel gene for SCA	The USA/2017	[53]

AD, alzheimer's disease; ALS, amyotrophic lateral sclerosis; IFN-*β*, interferon-*β*; MS, multiple sclerosis; PD, parkinson's disease; PLD, phospholipase D; SCA, spinocerebellar ataxia; and the USA, the United States of America.

## Data Availability

No underlying data was collected or produced in this study.

## Abbreviations

A*β*: Amyloid *β*
Ach: Acetylcholine
AD: Alzheimer disease
Arf: ADP-ribosylation factor
Arl2: Adenosine diphosphate-ribosylation factor-like protein 2
APP: Amyloid precursor protein
ALS: Amyotrophic lateral sclerosis
CNS: Central nervous system
EAE: Experimental allergic encephalomyelitis
ERK: Extraneous signal-regulated kinase
GPCRs: G protein-coupled receptors
GTPases: Guanosine triphosphatases
MAPK: Mitogen-activated protein kinase
MS: Multiple sclerosis
mTOR: Mammalian target of rapamycin
PA: Phosphatidic acids
PC: Phosphatidylcholine
PD: Parkinson's disease
PLD: Phospholipase D
SCA: Spinocerebellar ataxia
sph-1-P: Sphingosine-1-phosphate
Rac: Ras-related C3
Rho: Rhodopsin
Rho GDI: Rho-specific guanine nucleotide dissociation inhibitor
HKD: HXKX4DX6GSXN.

## Additional Points

*Limitations*. The development of PLD drugs is currently facing numerous challenges. First and foremost, the current challenge is to determine whether PLD inhibitors can be fully utilized to halt the progression of the disease in its acute stages and finding treatments with optimal pharmacokinetic properties. It is crucial to ascertain whether PLD inhibitors offer any advantages over existing treatments in terms of concerns about adverse effects. Second, there is currently limited information available regarding the specific role of each isoform and the molecular mechanisms that differ in their regulation of PLD isozyme synthesis. It remains uncertain whether PLD isozyme regulation occurs simultaneously or independently within the CNS. The choice between using a PLD monomer inhibitor or a dual PLD1/2 inhibitor may depend on the degree of PLD redundancy present in the process being inhibited. In EAE mice, there is a conspicuous consistency between the use of short-term PLD inhibitors and clinical improvement [80]. However, long-term inhibitor use may not yield many additional benefits. Further studies are needed to determine whether this finding can be used to direct therapeutic strategies. Third, it will be crucial to conduct and expand quantitative measurements of PA and its metabolic products in various disease states to determine how the misregulation of PLD1, PLD2, or any other enzyme involved in these diseases. An important point that needs to be addressed is whether PA primarily affects innate immunity mechanisms or if it serves a biophysical role.
